# Anatomical Treatment Strategies for Persistent Atrial Fibrillation with Ethanol Infusion within the Vein of Marshall—Current Challenges and Future Directions

**DOI:** 10.3390/jcm13195910

**Published:** 2024-10-03

**Authors:** Masaaki Yokoyama, Konstantinos Vlachos, Chizute Ogbedeh, Ciro Ascione, Christopher Kowalewski, Miruna Popa, Cinzia Monaco, Karim Benali, Kinan Kneizeh, Roberto Mené, Marine Arnaud, Samuel Buliard, Benjamin Bouyer, Romain Tixier, Rémi Chauvel, Josselin Duchateau, Thomas Pambrun, Frédéric Sacher, Mélèze Hocini, Michel Haïssaguerre, Pierre Jaïs, Nicolas Derval

**Affiliations:** 1 Hôpital Cardiologique du Haut-Lévêque, CHU de Bordeaux, 33604 Bordeaux-Pessac, France; 2 IHU LIRYC (L’Institut de Rythmologie et Modélisation Cardiaque), Université de Bordeaux, 33604 Bordeaux-Pessac, France; 3 School of Clinical Medicine, University of Cambridge, Cambridge CB2 1TN, UK; 4 Saint-Etienne University Hospital Center, Saint-Etienne University, 42100 Saint-Étienne, France

**Keywords:** anatomical approach, catheter ablation, persistent atrial fibrillation, the vein of Marshall

## Abstract

Currently, pulmonary vein isolation (PVI) is the gold standard in catheter ablation for atrial fibrillation (AF). However, PVI alone may be insufficient in the management of persistent AF, and complementary methods are being explored. One such method takes an anatomical approach—improving both its success rate and lesion durability may lead to improved treatment outcomes. An additional approach complementary to the anatomical one is also attracting attention, one that focuses on epicardial conduction. This involves ethanol ablation of the vein of Marshall (VOM) and can be very effective in blocking epicardial conduction related to Marshall structure; it is becoming incorporated into standard treatment. However, the pitfall of this “Marshall-PLAN”, a method that combines an anatomical approach with ethanol infusion within the VOM (Et-VOM), is that Et-VOM and other line creations are not always successfully completed. This has led to cases of AF and/or atrial tachycardia (AT) recurrence even after completing this lesion set. Investigating effective adjunctive methods will enable us to complete the lesion set with the aim to lower the rates of recurrence of AF and/or AT in the future.

## 1. Introduction

Radiofrequency (RF) catheter ablation is an established treatment method for rhythm control in symptomatic and drug-refractory atrial fibrillation (AF) patients [1,2]. In the latest ESC guideline [2], catheter ablation is recommended in patients with paroxysmal or persistent AF resistant or intolerant to arrhythmic drug therapy as class I. It is also newly recommended as a first-line option within a shared decision-making rhythm control strategy in patients with paroxysmal AF as class I by accumulating evidence. However, the position of catheter ablation as a first-line option in patients with persistent AF remains in class IIb.

Pulmonary vein isolation (PVI), aimed to eliminating the influence of ectopic beats. PVI in patients with PAF results in stable sinus rhythm in 87.8% of patients undergoing multiple procedures during 2-year follow-up [3], but only in 62.7% of patients during 10-year follow-up [4]. Furthermore, PVI is not an effective enough strategy for persistent AF (PsAF) patients. Several approaches have been utilized for the ablation for PsAF, such as the anatomical and electrogram-guided approaches; however, the best strategy for treating PsAF remains unknown. Tailored ablation approaches, including the aforementioned electrogram-guided approach, are effective at terminating AF even in the acute setting. This; however, has been associated with a high risk of subsequent organized atrial tachycardias (ATs). The results of the STAR-AF II trial showed no benefit in clinical outcome with such approaches beyond PVI [5,6]. A meta-analysis showed that the effectiveness of AF ablation for PsAF increases from 43% after one session to 69% after multiple sessions using several techniques [7]. Moreover, the durability of these lesion sets is also important. Regarding the anatomical approach, the efficacy of the modified Cox-Maze method is spectacular [8,9]. If the problem of low success rates and durability by percutaneous catheter ablation is resolved, the efficacy of the anatomical approach will improve as a consequence. One current challenge is achieving a complete block of the mitral isthmus (MI) line [10]. This is partly explained by the presence of epicardial bridges including the vein of Marshall (VOM) and the great cardiac vein (GCV)-coronary sinus (CS) [10,11,12,13]. Therein lies the motivation for VOM chemical ablation which is considered to be an effective method of targeting these epicardial bridges.

## 2. The Marshall Structure and Its Relationship with Atrial Arrhythmia

**Figure 1:**
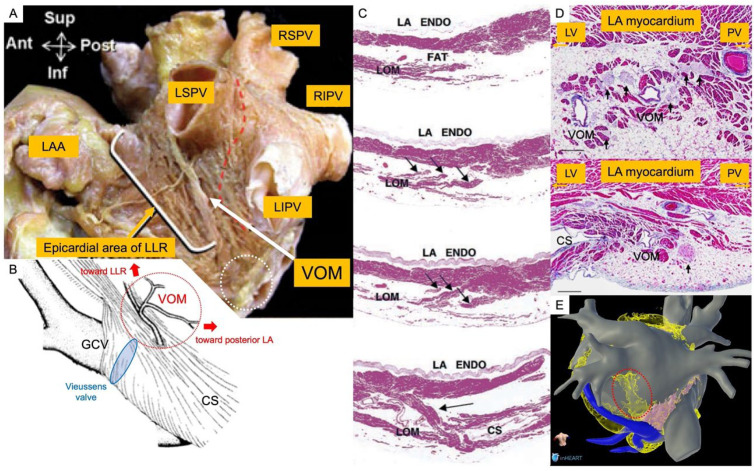
Marshall structure involving the VOM itself and surrounding structures. (**A**): Anatomical position of the vein of Marshall. It positioned in the epicardial area of the left lateral ridge (LLR) area that is between left atrial appendage and left pulmonary veins. (modified from Cabrera et al. [12]) (**B**): An enlarged view of the white dashed circle area of subfigure (A). The coronary sinus, the great cardiac vein and the VOM are surrounded by a complex course of the epicardial musculature. (modified from von Lüdinghausen et al. [11]) (**C**): Human serial sections of the ligament of Marshall (LOM). [16] It shows the LOM is isolated from the left atrial wall and there are multiple insertions into the left atrial wall and the coronary sinus. (**D**): Human histological slides of the LOM. Abundant nerve fibers (black arrow) around the VOM (above), and nerve ganglion (black arrow) are located in the vicinity of the CS juncture (below). These structures are very close to the Marshall structure. (modified from Makino et al. [14]) (**E**): An image of adipose tissue around the Marshall structure by CT reconstruction. Abundant adipose tissue (red dashed circle) exists around the Marshall structure. LAA = left atrial appendage; LSPV = left superior pulmonary vein; LIPV = left inferior pulmonary vein; RSPV = right superior pulmonary vein; RIPV = right inferior pulmonary vein; VOM = vein of Marshall; LLR = left lateral ridge; LA = left atrium; CS = coronary sinus; GCV = great cardiac vein; LOM = ligament of Marshall.

The Marshall structure contains the VOM itself, the Marshall bundle, autonomic nerves, and fat. Its intra-cardiac part is located on the epicardial aspect of the left lateral ridge (LLR) at variable distances from the endocardial surface of the same ridge (Figure 1A). In 70% of heart specimens the distance between the Marshall structure and the endocardial surface of the LLR was found to be <3 mm at the superior level of the ridge [12]. Despite this close proximity, radiofrequency energy cannot always penetrate through intervening tissue such as the surrounding fatty tissue. Small fibromuscular subepicardial bundles in this ridge connect the VOM with the left PVs and the left atrium (LA) free wall. These bundles increase in density near the opening of the VOM to the CS (Figure 1B). The epicardial fat pad of the LLR adjacent to the left PVs contains numerous parasympathetic ganglia and fibers of the autonomic nervous system. Makino et al. have already documented the correlation between the Marshall bundle and sympathetic nerve fibers within the PV-LA junctions (Figure 1D) [14]. By contrast, at the VOM-CS junction, regression of sympathetic fibers and an increase in parasympathetic ganglia are observed. This subtle variation in autonomic innervation patterns prompts further exploration of the functional implications of such regional heterogeneity and may potentially influence therapeutic strategies in the management of cardiac arrhythmias.

In clinical investigations, several insights associated with arrhythmia have been indicated (Table 1) [15,16,17,18,19,20]. Hwang et al., conducted meticulous assessments of electrical activity within the VOM in patients presenting with focal AF. Employing a quadripolar catheter, they achieved precise recordings by cannulating the VOM, offering valuable insights into the electrophysiological complexities associated with this anatomical region [15]. Kim et al. demonstrated that the ligament of Marshall consists of multiple myocardial tract insertions potentially establishing substrates for reentrant arrhythmias (Figure 1C) [16]. To investigate the neural properties of this region, Báez-Escudero et al. elucidated the complex composition of the VOM and revealed the presence of intrinsic cardiac nerves (ICN) which are intricately connected to the atrioventricular (AV) node. Their findings highlighted the pivotal role of these intrinsic neural elements in triggering AF. Furthermore, this study introduced a new treatment option and demonstrated that retrograde ethanol infusion into the VOM (Et-VOM) consistently abolished local ICN responses. This novel intervention not only highlighted the potential of targeted neuromodulation in the management of AF, but also established a reliable method for attenuating the specific neural triggers around the VOM [18,20]. These findings significantly contribute to our comprehension of the pathophysiology of exploring the Marshall bundle as a potential surrogate for atrial arrhythmogenesis.

## 3. The Importance of the Marshall Bundle in Complex Mitral Isthmus (MI) Linear Ablation

**Figure 2:**
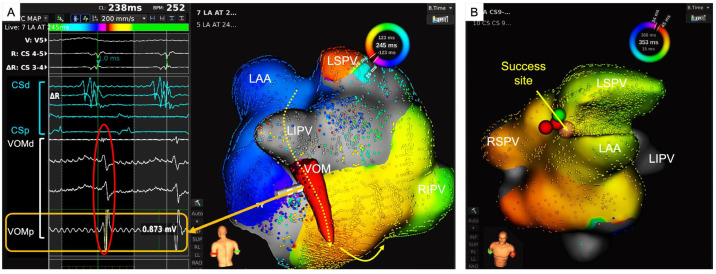
An example of VOM related atrial tachycardia. (**A**): Activation map during atrial tachycardia (AT) during a redo procedure after mitral isthmus linear ablation with potentials recorded by electrodes placed within the VOM. The potential recorded by the electrode placed in the VOM accounts for conduction during the blanking period of the tachycardia cycle within the VOM. (**B**): The success site for AT termination. Successful radiofrequency ablation was done at the high left lateral ridge area. LAA = left atrial appendage; LSPV = left superior pulmonary vein; LIPV = left inferior pulmonary vein; RSPV = right superior pulmonary vein; RIPV = right inferior pulmonary vein; VOM = vein of Marshall.

In a pivotal study by Jaïs et al., the efficacy of MI linear ablation was demonstrated, achieving an impressive 92% block rate in the acute phase through endocardial ablation combined with ablation within the CS [21]. However, its role in treatment of PsAF remains controversial. The results of the STAR-AF II trial, a large randomized controlled study, showed no benefit of linear ablation including an MI line. Of note, in a sub-study of untreated persistent AF (VENUS trial), combining catheter ablation and Et-VOM to obtain MI block gave better outcomes than without MI block [22]. It is important to note however that MI linear ablation can cause iatrogenic peri-mitral atrial tachycardia (PMAT), as exemplified in Figure 2 [23].

Conventional differential pacing using standard maneuvers to evaluate MI block has several limitations for assessing the achievement of true block [24,25]. Careful assessment using activation mapping with small multipolar electrodes and close spacing can better differentiate near-field from far-field potentials, revealing regions of slow residual conduction or epicardial connections from the CS musculature or the Marshall bundle [26]. Achieving MI block often requires ablation within the CS, resulting in reconnections that frequently demand further CS ablation [27].

Crucially, the creation of a transmural lesion to achieve MI block goes beyond technical proficiency; it requires the elimination of epicardial connections, including those associated with the Marshall bundle and the CS musculature. Vlachos et al. reported the role of Marshall bundle related atrial arrhythmias, including MI-dependent AT from Marshall bundle and/or Marshall bundle related reentries, constituting up to 30.2% of left ATs following AF ablation [28]. Et-VOM was popularized by Valderrábano et al. who pioneered this method. It has emerged as an effective strategy for achieving MI block and addressing PMAT [29,30,31,32,33,34]. According to the latest expert consensus, Et-VOM is now considered as “May be appropriate TO DO” for achieving block in the lateral MI in patients with mitral annular flutter [1].

## 4. Recent Studies on the Role of Et-VOM as Treatment of AF

As aforementioned, the VOM is implicated in AF. In recent studies on the role of Et-VOM, there has been a wealth of reports evaluating the effectiveness of catheter ablation (CA) + Et-VOM as a treatment paradigm in AF or AT patients. A landmark randomized controlled trial, the VENUS trial—a multicenter study, established the superiority of CA + Et-VOM over CA alone. Results of this study indicated a higher freedom from AF at 6 and 12 months after a single procedure (49.2% vs. 38%; *p* = 0.04) and after multiple procedures (65.2% vs. 53.8%; *p* = 0.04) in the CA + Et-VOM group than the CA alone group [33]. Recent meta-analyses including this study revealed the effectiveness of adjunctive Et-VOM, reducing AF/AT recurrence rates and successfully achieving acute MI bidirectional block without increasing adverse events rates [35,36,37,38].

Additionally, in PsAF patients with hypertrophic cardiomyopathy, adjunctive Et-VOM has been demonstrated to significantly reduce AF/AT recurrence after 12 months (84.6% vs. 65.2%; *p* = 0.03) [39]. This collective evidence highlights the efficacy of CA + Et-VOM as a versatile treatment method for diverse AF patient populations.

### 4.1. What Strategy of Catheter Ablation Should Be Combined with Et-VOM?

Despite numerous reports on the effectiveness of adjunctive Et-VOM and various proposals for CA strategies combined with Et-VOM, a universally accepted standard method has not yet been established. In the VENUS trial, MI ablation was notably more prevalent in the CA + Et-VOM group (85.4%) compared with the CA alone group (72.2%), indicating a significant difference in the frequency of implementation (*p* = 0.003) [33]. Taking this point into consideration, it is possible that the lack of mitral block may have contributed to the clinical outcomes. Actually, in the sub-analysis of this study, in patients with perimitral block, freedom from AF or AT was reached 54.3% of the time after CA + Et-VOM and 37% after CA alone [22]. In this trial, various other lesion sets were performed, including posterior wall isolation and ablation of complex potentials, and it cannot be denied that these differences in the frequency of attempt may have influenced the differences in clinical outcomes.

**Figure 3:**
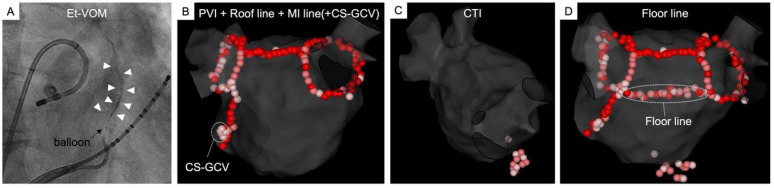
The lesion setting of the Marshall-PLAN. (**A**): Retrograde ethanol infusion within the VOM. (**B**): Wide antral pulmonary vein isolation (PVI) by RFA—an anatomical roof line connecting 2 encircling PVI lines and the mitral isthmus line from the left inferior PV to the mitral annulus. (**C**): Radiofrequency ablation of the cavotricuspid isthmus. (**D**): Radiofrequency ablation of an additional floor line. If first pass block of roof line cannot be achieved, ablation of the floor at the junction of the dome with the posterior wall is done as a second line. Et-VOM = ethanol infusion within the vein of Marshall; PVI = pulmonary vein isolation; MI = mitral isthmus; CTI = cavotricuspid isthmus; CS = coronary sinus; GCV = great cardiac vein.

The anatomical approach employed in the “Marshall-PLAN” lesion set (Marshall bundle elimination, Pulmonary vein isolation, and Line completion for ANatomical ablation of persistent atrial fibrillation), as shown in Figure 3, is a simplification of the “Cox-Maze” set. It involves: (1) Et-VOM primarily aimed at facilitating mitral isthmus block, (2) wide antral PVI, (3) a roof line connecting the 2 encircling PVI lines, (3) an MI line from the left inferior PV to the mitral annulus, (4) a cavotricuspid isthmus (CTI) line [40,41]. This lesion set concept also respects normal atrial activation in sinus rhythm [42]. Notably, we do not include the anterior MI line in our clinical practice due to the associated left atrial appendage (LAA) activation delay [43] and the thickness of the Bachman bundle making it challenging to achieve transmural block using solely endocardial ablation [44].

### 4.2. Methodology of the Marshall-PLAN

Our methodology of the Marshall-PLAN has been previously reported in detail [40,41]. Below is a brief description of the method.

General principles:

The procedure is performed under conscious sedation or general anesthesia using 3D electroanatomic mapping system. A steerable long sheath is systematically used. Radiofrequency ablation is performed in a point-by-point fashion. An RF current is delivered for 8–30 s (at 20–25 W for the CS, 35–50 W for the posterior wall, 40–50 W for all other atrial sites). Automated lesion annotation is performed.

Et-VOM:

Et-VOM is performed prior to RF ablation. After CS cannulation with the sheath, a left internal mammary artery (LIMA) catheter is inserted. Angiographic contrast is injected near the Vieussens valve to localize the VOM. A preloaded angioplasty balloon with an angioplasty guidewire is advanced in the VOM near its ostium. A total amount of 6–10 mL 96% ethanol is infused in three separate 1-minute injections. Repeat angiograms are performed after each injection to confirm balloon stability, visualization of distal VOM arborization, absence of contrast leakage back to the CS, absence or limited dissection of the VOM and progressive appearance of tissue contrast staining.

PVI:

Wide antral circumferential ablation (WACA) is performed.

Lines:

Three lines are systematically created across the 3 main anatomical isthmuses: (1) a roof line connecting the 2 encircling PVI lines; (2) a mitral line from the left inferior PV to the mitral annulus; and (3) a CTI line. If prior Et-VOM at the posterolateral MI region is reflected on the 3D map, RF application to the same area to create the MI line will be intentionally minimized. Additionally, CS and great cardiac vein RF ablation targeting epicardial muscular bundles is systematically performed. This ablation is performed where facing the MI line created. If there are sharp near-field signals at the free wall, the free wall itself is also ablated. Ablation is performed in AF if sinus rhythm has not been restored before creating linear lesions. If AF persists at the end of linear ablation, electrical cardioversion is performed to restore sinus rhythm. Regarding the roof line, when first-pass block cannot be achieved, a second line involving ablation of the floor line at the lower border of the inferior PVs is implemented to address dome-isthmus transection [41].

### 4.3. Pitfalls and Additional Advantages of the Marshall-PLAN Lesion Set

#### 4.3.1. Et-VOM

Considering the variability in the branching pattern of the VOM among individuals, the impact of Et-VOM may differ accordingly [45]. The impact of Et-VOM frequently extends to the part of the MI near the left inferior PV (LIPV) and around the LIPV itself [46], resulting in reported benefits for left PV isolation [47,48]. Furthermore, addressing the posterior vein branching from the VOM allows for minimizing ablation on the posterior side and eliminating epicardial fibers bridging the floor line as well as MI line [49], potentially reducing esophageal injury [50]. Notably, while it has been reported that the scar area immediately after Et-VOM shrinks after 1 h [51], there is no obvious reduction in the scar area when comparing the results from after the initial procedure and during the redo procedure [52]. Nevertheless, we consistently create complete RFA linear lesions even in the scar area immediately after Et-VOM, recognizing the possibility that Et-VOM may not fully penetrate the endocardial layer.

**Figure 4:**
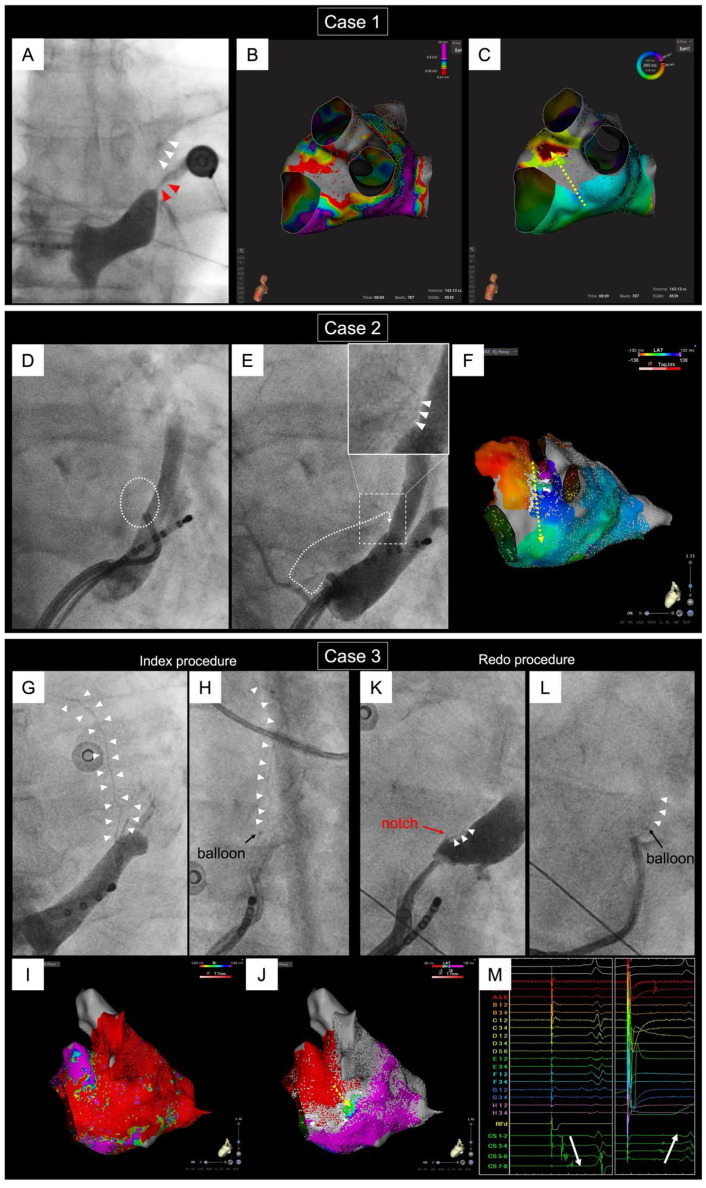
Difficult cases of ethanol infusion within the VOM in redo procedures. Case 1. An example of CS stenosis and VOM occlusion caused by previous multiple radiofrequency ablation in the CS-GCV. (**A**): Coronary sinus venography during the redo procedure. Previous radiofrequency ablation caused CS stenosis (red arrows) and VOM occlusion. The VOM (white arrows) is visualized by flow from the distal part. (**B**,**C**): Voltage map and activation map in the left atrium delineated at the beginning of the redo procedure. The posterior mitral isthmus has been blocked endocardially but reconnects epicardially. However, because we could not access the VOM, we had to perform RFA with the endocardial approach. Case 2. An example of VOM occlusion during the redo procedure after Et-VOM was performed during the index procedure. (**D**): Contrast medium infusion at the ostium of the VOM. The VOM is not visualized in this venography. (**E**): Retrograde contrast medium infusion within the posteroseptal vein arising from close to the CS ostium. The VOM is visualized by collateral flow from this vein. (**F**): Activation map in the LA delineated at the beginning of the redo procedure. The mitral isthmus has been blocked endocardially but reconnects epicardially. However, because we could not access the VOM, we had to perform RFA with the endocardial approach. Case 3. An example of VOM occlusion of its main trunk during the redo procedure after Et-VOM during the index procedure. (**G**): Retrograde venography of the VOM during the index procedure. The VOM branched near the ostium of itself. (**H**): Et-VOM of the main trunk during the index procedure—posterior mitral block was achieved. (**I**,**J**): Voltage map and activation map in the left atrium delineated at the beginning of the redo procedure. The posterior mitral isthmus has been blocked endocardially but reconnects epicardially. (**K**): Retrograde venography of the VOM using a Swan-Ganz catheter. This was done because it was difficult to visualize the VOM using IMA catheter due to previous Et-VOM and/or ablation in the CS-GCV. The main trunk of the VOM was seen only as a “notch”. However, the branch of the VOM was identified. (**L**): Et-VOM of the branch of the VOM. (**M**): The sequence of activation in the CS under pacing from the anterior side of the posterior mitral line was changed and mitral isthmus block was achieved after Et-VOM.

**Figure 5:**
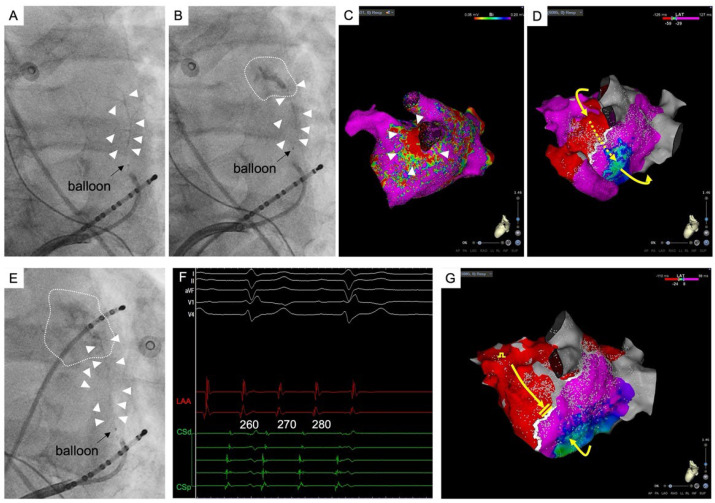
Redo Et-VOM due to reconnection of VOM conduction after index Et-VOM during the same procedure. (**A**,**B**): Index Et-VOM attempt. It was successfully done with 10 cc alcohol. (**C**): A voltage map immediately after the index Et-VOM attempt showing its typical effect. (**D**): An activation map in the left atrium and the coronary sinus during the atrial tachycardia (AT) that occurred after all lesions in the Marshall-PLAN were created. It was suspected that AT was related to the VOM regaining conduction ability based on this map. (**E**): Redo Et-VOM attempt—there is a great staining lesion along the distal part of the VOM. (**F**): During Et-VOM, AT terminated immediately. (**G**): An activation map under pacing from the left atrial appendage after the redo Et-VOM attempt. Posterior mitral isthmus block was successfully achieved after redo Et-VOM.

In order to fully reap the benefits of Et-VOM, it must be successfully completed. Our method of Et-VOM has been previously reported in detail [40]. Although the initial success rate of Et-VOM was approximately 92% in our institute’s preliminary data, a meta-analysis revealed a slightly reduced rate of 86.7% (95% CI 81.9–91.4%) [35]. Of note is the discernible learning curve influencing the success rate of Et-VOM [53], and the success rate in cannulating the VOM was clearly lower in low volume centers than in high volume ones (67.3% vs. 90.2%; *p* < 0.001) [22]. The primary cause of Et-VOM failure predominantly stemmed from nonidentification [53], a factor possibly linked to the procedural learning curve. Multivariable analysis has pinpointed previous ablation in the CS as the sole predictor for nonidentification [53]. Indeed, previous ablation in the CS causes CS stenosis in 12% of redo patients [54]. Interestingly, instances have been observed where successful Et-VOM in a previous procedure did not preclude the emergence of AT, nor a gap in the MI involving the Marshall bundle, during a subsequent redo procedure [55]. Remarkably, the VOM could not be identified in 74.4% of such cases [55]. In addition to the impact of previous CS ablation, there exists the possibility that cannulation and Et-VOM become unattainable due to dissection incurred during previous procedures, as well as thrombus formation and stenosis within the VOM arising from previous Et-VOM (Figure 4). Consequently, ensuring a robust and enduring Et-VOM completion in the initial procedure is paramount, as shown in Figure 5.

Low-voltage areas induced by Et-VOM can have various sizes which probably depend on the arborization pattern of the VOM [45]. Lam et al. described a significant correlation between the extent of the low-voltage area and the volume of ethanol injected (*p* = 0.03) [31], highlighting the important role of infusion parameters in influencing the outcome of the procedure. Traditionally, most electrophysiologists have adhered to a standard injection range of 2–4 mL of ethanol within the VOM, guided by previous publications [30,33]. However, the Marshall-PLAN adopts a more liberal approach with a total infusion volume of 10 mL administered in three doses of 3.3 mL each [40,41]. This departure from the conventional approach prompts further investigation into the knock-on effects on lesion characteristics and procedural efficacy. The variation in ethanol infusion parameters, including the volume per dose and infusion duration, is evident across existing reports, highlighting the absence of a standardized protocol within the field (Table 2) [30,33,34,40,56,57]. Another challenging and debated aspect of the Et-VOM procedure centers on the optimal placement of the balloon during ethanol infusion—i.e., distal or proximal. These important elements are currently under investigation and are necessary for refining and standardizing Et-VOM procedures, ensuring optimal clinical outcomes.

#### 4.3.2. Lines

MI line:

A systematic approach to MI line creation involves radiofrequency ablation (RFA) which targets epicardial muscular bundles in the CS and GCV due to their frequent myocardial sleeves [11], necessitating additional RF application during redo procedures [27]. Moreover, even after ablating the CS-GCV anchored wall, a gap at the CS-GCV free wall, is occasionally observed (11% among the patients whose mitral isthmus block was obtained by CS-GCV endovascular ablation) [10]. Therefore, it is occasionally necessary to perform additional free wall ablation.

Roof line:

Because of its superior position with respect to the chest, the anatomical isthmus between the 2 sets of PVs is referred by anatomists as the “dome” rather than the “posterior wall” (PW). The latter terminology is used for the area joining the lower border of the inferior PVs to the vestibule surrounding the mitral annulus. Therefore, the term “dome transection” is used to describe a conduction block across that anatomical isthmus [58].

When first-pass block cannot be achieved, a second line involving ablation of the floor at the lower border of the inferior PVs is implemented to address dome isthmus transection [41]. Evaluation of roof line block based solely on the recording of double potentials along the entire roof line [59] may be misleading in cases of endocardial-epicardial breakthroughs [58]. The unique structural characteristics of the dome contribute to the complexities of this anatomical structure and offers an ideal bypass for the roof line. These include: its thickness, the presence of septopulmonary bundles—first reported by Papez as an epicardial layer [60]—and the interposition of adipose tissue which creates a clear demarcation between the epicardial and endocardial layers [44,58,61]. Additionally, floor line block is achieved more frequently than roof line block (85% vs. 67%; *p* = 0.049), as the junction of the dome with the posteroinferior wall tends to be thinner with less fat [58]. Consequently, we presently choose to shift to creating a floor line to block the dome isthmus, rather than insisting on completing the anatomical roof line.

#### 4.3.3. LA Function

It has been pointed out that additional LA ablation beyond PVI is related to stiff LA syndrome [62]. However, the relationship between Et-VOM and stiff LA syndrome has not yet been described. In a Marshall-PLAN study, analysis of A-wave velocities based on echocardiography data revealed significant improvements in LA function by 12 months [40]. These findings indicate that the Marshall-PLAN ablation lesion set does not inadvertently ablate functionally important areas of the LA. Nonetheless, if other additional approaches beyond the standard Marshall-PLAN ablation lesion set are performed, the impact on LA function may differ from the results above. In either strategy, it may be important to evaluate not only using A-wave velocity but also using advanced echography indicators [63], as a means of determining whether atrial function can be maintained postoperatively.

### 4.4. Outcomes of the Marshall-PLAN and Those of Similar Strategies

Our Marshall-PLAN initiative resulted in favorable outcomes, demonstrating a 72% freedom from AF/AT recurrence rate at 12 months post-initial procedure in PsAF patients. When including the second procedure, this rate rose to an impressive 89% [40].

Recently, Lai et al. reported findings from their randomized single-center study utilizing the upgraded “2C3L” method which lesion set is quite similar to the Marshall-PLAN [34]. They revealed that during a 12-month follow-up, 87.9% of patients in the group that created 2C3L with CA + Et-VOM had no AF/AT, whereas 64.8% of patients in the group that created 2C3L with CA alone (*p* < 0.001), showing the superiority of CA + Et-VOM. These insights will contribute to the ongoing research regarding optimal ablation strategies in the evolving landscape of AF treatment. Nevertheless, when comparing the results of various studies conducted with a design that combines Et-VOM with catheter ablation (Table 3) [30,33,34,40,56,57], it is important to consider not only the ablation strategy, but also the implementation rate and success rate of various lines, as well as how to evaluate the bidirectional block of these lines.

## 5. Enhancing the Marshall-PLAN Protocol: Strategies for Improved Outcomes and Future Prospects

### 5.1. Pulsed Field Ablation; A Novel Ablation Modality Using an Electric Field

Pulsed field ablation (PFA) is a novel modality using an electric field which has been shown to be safe and efficient [64,65,66]. This novel modality is mainly used for PVI but also for creating linear lesions such as for posterior wall isolation [67,68] and mitral isthmus block [69]. However, the durability of these lesions, particularly regarding the MI line with or without Et-VOM, is not well-understood. Additionally, creating the MI line using PFA can sometimes cause coronary spasm [70]. Even if PFA achieves a durable block of the MI line without adjunctive Et-VOM, it still may not be as effective as RFA combined with Et-VOM as PFA does not permanently ablate the surrounding nerves [71].

### 5.2. Recurrence Patterns Post-Marshall-PLAN Lesions and Additional Treatments to Consider

#### 5.2.1. LA Anterior Scar Related AT

Some supplemental approaches, in conjunction with PVI, left atrial anatomical lines, and Et-VOM, have been proposed to address the challenge of residual AF recurrence. Exploring and refining these additional strategies may hold the key to further optimizing the Marshall-PLAN protocol for enhanced effectiveness in managing complex arrhythmias. Within the cohort of patients who underwent a comprehensive Marshall-PLAN protocol during the index procedure and subsequently required a redo procedure due to recurrent AF/AT, 52% experienced AF recurrence, while 48% experienced AT recurrence [41]. High resolution mapping during the redo procedure, focusing on 20 patients with stable AT, revealed that 65% had gap-related AT within the Marshall-PLAN lesion set. The remaining 35% had scarring of the anterior LA. These findings suggest that once a durable Marshall-PLAN lesion set is created, there is a potential for scar-related reentry in the anterior LA. However, if an anterior mitral line—as treatment of scar-related AT in the anterior LA—is created following a complete Marshall-PLAN lesion set, it may undesirably lead to isolating the left half of the LA anterior wall including the LAA. To combat this, an ablation line halfway from the anterior scar area to the anterior mitral annulus can be created rather than a complete anterior mitral line. It is unclear whether such treatment should be added to initial ablation for all patients with anterior scars.

#### 5.2.2. Posterior Wall Isolation (PWI) as an Adjunct to the Marshall-PLAN Lesion Set

Ishimura et al. devised a comprehensive ablation strategy involving PVI, a MI line, Et-VOM, superior vena cava isolation and PWI which demonstrated notable success. Remarkably, about 75% of patients remained free from AF/AT recurrence during a 1-year follow up period [57]. This highlights the potential effectiveness and importance of including PWI in the ablation strategy for achieving sustained rhythm control in patients with AF. However, they failed to show the added benefit of PWI as they did not compare the recurrence rate of the strategy with PWI to without it. This may be due to the fact that PWI does not have high lesion durability, as shown in the results of the CAPLA study (31.2%) [72]. If durable PWI can be created, for example using PFA (85%) [68], combined with the durable Marshall-PLAN lesion set, outcomes may improve further.

### 5.3. The Present State and the Future Direction of Anatomical Approach Strategies Using t-VOM

**Figure 6:**
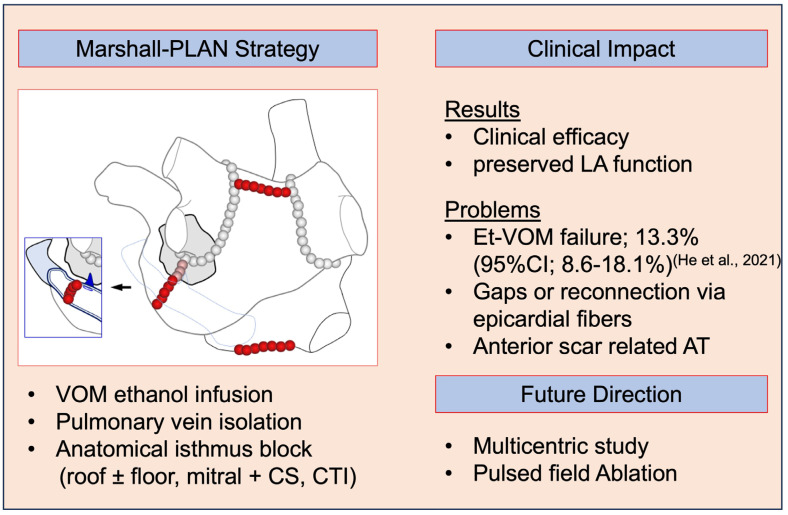
**(Central illustration).** Clinical impact and the future direction of anatomical approach strategies using Et-VOM [35].

The clinical impact and future directions of anatomical approach strategies using Et-VOM are summarized in Figure 6 as a central illustration. The Marshall-PLAN study reported good clinical outcome with sparing LA function. However, this study was a single arm, non-randomized study. Mono- and multicentric randomized controlled trial are currently underway and might fill the evidence gap. One of the main problems of this strategy is whether our current capabilities allow us to easily create complete and durable linear lesions. The recent rise of pulsed-field ablation might solve that issue as suggested by recent study [67,68,69]. However, future studies are required to confirm the long-term results.

## 6. Conclusions

Although the “Marshall-PLAN” proves to be valuable in clinical practice, achieving a durable lesion set remains challenging, especially after a single procedure. This is even despite creating a floor line when a successful roof line lesion is not possible. Given the complexities involved in accessing the vein of Marshall during a redo procedure, it is imperative to identify the most effective method of eliminating Marshall bundle epicardial connections during the initial attempt. Moreover, even with the achievement of these durable lesion sets, in a subset of cases atrial fibrillation/atrial tachycardia recurrence still occurs. The pursuit of refining the Marshall-PLAN protocol demands a comprehensive understanding of the challenges faced during the lesion set process and a strategic approach to improve the overall effectiveness of the ablation procedure.

## Figures and Tables

**Table 1:** The relationship between the Marshall structure itself and atrial arrhythmia. AF = atrial fibrillation; LOM = ligament of Marshall; CS = coronary sinus; VOM = vein of Marshall; LA = left atrium; CS = coronary sinus.

Study (yr.)	Animal/Human	Number of Patients	Arrhythmia Mechanism
Hwang, et al. (2000) [15]	Human	28	Focal AF originated from the muscle bundle within the LOM
Kim, et al. (2000) [16]	Human (postmortem)	7	Possibility of reentry due to multiple discrete myocardial tract insertions into the left atrial free wall and the CS
Morita, et al. (2012) [17]	Animal	16	Reentry due to myocardial connection of the VOM with the LA and the VOM with the CS
Valderrábano, et al. (2009) [18]	Animal and Human	17 (Animal)/6 (Human)	First report of ethanol infusion in the VOM Evaluation of the impact on arrhythmia mechanism
Choi, et al. (2010) [19]	Animal	6	Paroxysmal AF preceded by intrinsic cardiac nerve activity (including the LOM nerve activity)
Báez-Escudero, et al. (2014) [20]	Human	133	AF triggered by intrinsic cardiac nerves involved in the Marshall structure

**Table 2:** Differences in Et-VOM methodology (volume of ethanol per infusion, number of infusions, duration of ethanol infusion per infusion and balloon position during ethanol infusion) in each literature. VOM = vein of Marshall; NR = not reported.

Study (yr.)	Volume of Ethanol per Infusion (Total)	Duration of Ethanol Infusion per Infusion	Number of Ethanol Infusions	Balloon Position during Ethanol Infusion
Valderrábano, et al. (2020) [33]	1 mL	>2 min	up to 4	changing from distal to proximal gradually, (retracted ≤ 1 cm, respectively, until the balloon is at the ostium of VOM)
Liu, et al. (2019) [30]	1 mL	1 min	2–4	NR
Derval, et al. (2020) [40]	≤3 mL (6–10 mL)	1 min	3	proximal VOM
Kawaguchi et al. (2019) [56]	1.5–2.0 mL	90 s	1–3	changing from distal to proximal gradually (until the balloon is at the ostium of VOM)
Ishimura et al. (2023) [57]	3 mL; distal: 2 mL; proximal (5 mL)	NR	2	the most distal site and the most proximal site
Lai et al. (2021) [34]	2–4 mL (max 12 mL)	NR	NR	distal and proximal and/or middle site

**Table 3:** Characterization of various studies designed to combine ethanol infusion within the vein of Marshall with catheter ablation. PsAF = persistent atrial fibrillation; PAF = paroxysmal atrial fibrillation; AT = atrial tachycardia; Et-VOM = ethanol infusion within the vein of Marshall; PVI = pulmonary vein isolation; PWI = posterior wall isolation; MI = mitral isthmus (line); CFAE = complex fractionated atrial electrograms; RFA = radiofrequency ablation; CTI = cavotricuspid isthmus (line); SVCI = superior vena cava isolation; 2C3L = bilateral circumferential pulmonary vein antrum isolation and three linear ablation lesion sets; NR = not reported; CS = coronary sinus; LAA = left atrial appendage; LA = left atrium; SA = stimulus-atrial; CSd = distal coronary sinus; CSp = proximal coronary sinus; CA = catheter ablation; PMAT = perimitral atrial tachycardia; RSPV = right superior pulmonary vein.

Study (yr.)	Study Population (AF Type)	Ablation Strategy		Et-VOM Success Rate	Method of Checking the Lines (MI Line)
Valderrábano, et al. (2020) [33]	Drug refractory symptomatic PsAF (n = 350)	Et-VOM group: Et-VOM + PVI + discretion of the operator (PWI, MI, CFAEs) (n = 185) RFA group: PVI + discretion of the operator (PWI, MI, CFAEs) (n = 158)		155/185 (83.7%)	bidirectional block based on differential pacing (Bidirectional block refers to elimination of electrical propagation across the mitral isthmus in both directions)
Liu, et al. (2019) [30]	Drug refractory PsAF (n = 254)	Et-VOM + PVI ± substrate ablation group: Et-VOM + PVI + Lines (MI + Roof + Septal + CS) ± CFAEs + etc. (n = 32) PVI+ substrate ablation group: PVI + Lines (MI + Roof + Septal + CS) ± CFAEs + etc. (n = 139 [including Et-VOM failed]) (or PVI alone group (n = 83)		32/41 (78%)	bidirectional block (detail: NR)
Derval, et al. (2020) [40]	PsAF (n = 75)	Single arm: Et-VOM + PVI + MI + Roof + CTI		69/75 (92%)	bidirectional block (detail: NR)
Kawaguchi et al. (2019) [56]	PAF (n = 50) (60%), PsAF (n = 34) (40%)	Single arm: Et-VOM + PVI (Cryo; 87%, RFA; 13%) +MI		84/115 (73%)	bidirectional block based on differential pacing (detail: NR)
Ishimura et al. (2023) [57]	PAF (n = 60) (14%), PsAF (n = 345) (83%), AT (n = 8) (2%)	Et-VOM group: Et-VOM + PVI + PWI + MI + SVCI + CTI (n = 177) RFA group: Et-VOM + PVI + PWI + MI + SVCI + CTI (n = 236 [including Et-VOM failed])		177/261 (67.8%) success population including partial success cases	bidirectional block based on differential pacing (detail: NR)
Lai et al. (2021) [34]	Drug refractory PsAF (n = 191)	Et-VOM + 2C3L group: Et-VOM + PVI +MI +Roof +CTI (n = 66) 2C3L group: PVI +MI +Roof +CTI (n = 125)		53/66 (80.3%)	double potential along the ablation line is used for a preliminary screening of conduction block bidirectional block confirmed by; (1) proximal-to-distal CS activation pattern when pacing at the LAA and left lateral ridge (2) the activation is from the LA lateral wall to the ablation line when pacing at the distal CS or SA interval at LAA is longer when pacing at CSd than pacing at CSp
**Study (yr.)**	**Implementation Rate and Success Rate of Lesion Creations**		**AF Recurrence (Follow Up Duration)**		**Findings in Redo Procedure**
Valderrábano, et al. (2020) [33]	**Et-VOM + RFA vs. RFA alone (as randomized)** Implementation rate: MI: 158/185 (85.4%) vs. 114/158 (72.2%) (Ablation in the CS: 55/185 [29.7%] vs. 74/158 [46.8%]) PWI: 123/185 (66.5%) vs. 118/158 (74.7%) CFAEs: 167/185 (90.3%) vs. 151/158 (95.6%) Acute success rate: MI: 137/158 (86.7%) vs. 81/114 (71.0%)		90 days after index procedure (excluding death and missing data) Et-VOM + RFA (as randomized): 77/168 (45.8%) RFA (as randomized): 82/142 (57.7%) Et-VOM + RFA (as treated): 62/142 (43.7%)		NR
Liu, et al. (2019) [30]	**Et-VOM (only including Et-VOM success cases) + PVI ± substrate ablation vs. PVI+ substrate ablation** Implementation rate: Lines: 27/32 (84.4%) vs. 103/139 (74.1%) (CFAEs:9/32 [28.1%] vs. 92/139 [66.2%]) Acute success rate: NR		Et-VOM (only including Et-VOM success cases) + RFA vs. RFA alone 9/32 (28.1%) (AT; 4) vs. 83/139 (59.7%) (AT; 18) (follow up duration: 3.9 ± 0.5 yrs)		NR
Derval, et al. (2020) [40]	Implementation rate: MI (100%), Roof (100%), CTI (100%) Acute success rate: MI 71/75 (95%), Roof 74/75 (99%), CTI 74/75 (99%)		21/75 (28%) (AT; 9) (follow up duration: 12 months) 8/75 (11%) (After 1 or 2 procedures)		**Redo procedure; 19 cases** (In all cases, at least 1 reconnection was identified) (Left PV 7, Right PV 15, Roof line 13, MI line 12, CTI 7) >>> 13/19 (68%) had a successful Et-VOM 6/19 still exhibited a reconnected mitral line after Et-VOM: (4 at the epicardial aspect [CS ablation], 2 at the endocardial aspect [mitral annulus])
Kawaguchi et al. (2019) [56]	Implementation rate: MI (100%) (only among Et-VOM success cases) Acute success rate: MI: 78/84 (92.9%)		22/84 (PAF 9, PsAF 13) (26.2%) (follow up duration: NR)		**Redo procedure; 17 cases** 6/15 MI line reconnected (except for 2/17 of MI block failure at the first procedure) (PMAT 2) >>> 3/8 required re-ablation at the endocardial aspect alone, 5/8 required ablation at the epicardial aspect (CS ablation)
Ishimura et al. (2023) [57]	**Et-VOM ≥ 5mL vs. Et-VOM < 5 mL vs. Et-VOM(-)** Implementation rate: MI (100%), PWI (100%), SVCI (NR), CTI (NR) Acute success rate: MI: 95/106 (90%) vs. 66/71 (93%) vs. 210/236 (89%) PWI: 105/106 (99%) vs. 71/71 (100%) vs. 236/236 (100%)		Et-VOM ≥ 5 mL vs. Et-VOM < 5 mL vs. Et-VOM(-) 26/106 (24%) (AT; 10) vs. 28/71 (40%) (AT; 12) vs. 71/236 (30%) (AT; 24) (follow up duration: NR)		**Redo procedure; 74 cases** Et-VOM ≥ 5mL; 17(AT; 9) MI-dependent AT, 1; roof-dependent AT, 1; anterior wall AT, 3; focal AT from the roof area, 1; focal AT from the LA septum area, 1; no inducibility, 2 Et-VOM < 5 mL; 18(AT; 8) MI-dependent AT, 3; anterior wall AT, 1; CTI-dependent AT, 1; no inducibility, 3 Et-VOM(-); 38(AT; 17) MI-dependent AT, 3; roof-dependent AT, 1; anterior wall AT, 4; focal AT from the VOM, 2; focal AT from the mitral annulus, 1; gap re-entry associated with the reconnected left PV, 1; focal AT or localized re-entry in the SVC, 1; no inducibility, 4 MI reconnection rate; NR PWI reconnection rate: Et-VOM ≥ 5 mL vs. Et-VOM < 5 mL vs. Et-VOM(-); 9/17 (53%) vs. 10/19 (53%) vs. 25/38 (66%)
Lai et al. (2021) [34]	**Et-VOM + 2C3L vs. 2C3L** Implementation rate: MI (100%), Roof (100%), CTI (100%) Acute success rate: MI: 63/66 (95.5%) vs. 101/125 (80.8%) Roof: 66/66 (100%) vs. 125/125 (100%) CTI: 66/66 (100%) vs. 124/125 (99.2%)		Et-VOM + 2C3L vs. 2C3L 8/66 (12.1%) (AT; 2) vs. 44/125 (35.2%) (AT; 8) (follow up duration: 12months)		**Redo procedure; 16** Et-VOM + 2C3L; 4(AT; 3) PMAT, 2 (required re-ablation at the annulus side of MI) gap of RPV—related AT, 1 Et-VOM; 12(AT; 8) PMAT, 3; roof-dependent AT, 2; gap of RSPV—related AT, 2; Scar—related AT, 1

## Data Availability

No new data were created or analyzed in this study. Data sharing is not applicable to this article.
